# Acute effects of game-based biofeedback training on trunk motion in chronic low back pain: a randomized cross-over pilot trial

**DOI:** 10.1186/s13102-022-00586-z

**Published:** 2022-11-13

**Authors:** Juliane Mueller, Daniel Niederer, Sarah Tenberg, Lukas Oberheim, Alina Moesner, Steffen Mueller

**Affiliations:** 1 Department of Computer Science – Therapy Sciences, Trier University of Applied Sciences, Trier, Germany; 2 Department of Sports Medicine and Exercise Physiology, Institute of Occupational, Social and Environmental Medicine, Goethe University, Frankfurt, Germany

**Keywords:** LBP, Non-specific, Rehabilitation, Exergame, Health technologies

## Abstract

**Background:**

Improving movement control might be a promising treatment goal during chronic non-specific low back pain (CLBP) rehabilitation. The objective of the study is to evaluate the effect of a single bout of game-based real-time feedback intervention on trunk movement in patients with CLBP.

**Methods:**

Thirteen CLBP patients (8female;41 ± 16 years;173 ± 10 cm;78 ± 22 kg) were included in this randomized cross-over pilot trial. During one laboratory session (2 h), participants performed three identical measurements on trunk movement all including: first, maximum angle of lateral flexion was assessed. Secondly, a target trunk lateral flexion (angle: 20°) was performed. Main outcome was maximum angle ([°]; MA). Secondary outcomes were deviation [°] from the target angle (angle reproduction; AR) and MA of the secondary movement planes (rotation; extension/flexion) during lateral flexion. The outcomes were assessed by an optical 3D-motion-capture-system (2-segment-trunk-model). The measurements were separated by 12-min of intervention and/or resting (randomly). The intervention involved a sensor-based trunk exergame (guiding an avatar through virtual worlds). After carryover effect-analysis, pre-to-post intervention data were pooled between the two sequences followed by analyses of variances (paired t-test).

**Results:**

No significant change from pre to post intervention for MA or AR for any segment occurred for the main movement plane, lateral flexion (p > .05). The upper trunk segment showed a significant decrease of the MA for trunk extension/flexion from pre to post intervention ((4.4° ± 4.4° (95% CI 7.06–1.75)/3.5° ± 1.29° (95% CI 6.22–0.80); p = 0.02, d = 0.20).

**Conclusions:**

A single bout of game-based real-time feedback intervention lead to changes in the secondary movement planes indicating reduced evasive motion during trunk movement.

*Trial registration* No. DRKS00029765 (date of registration 27.07.2022). Retrospectively registered in the German Clinical Trial Register.

## Introduction

With a lifetime prevalence of about 61% and frequently leading to disability in 10% to 15% of all patients affected, chronic non-specific low back pain (CLBP) is a major burden on health systems of western societies [1, 2].

The comparison of trunk kinematics and posture between patients with CLBP and healthy participants may be helpful in retrieving factors associated with the development, persistence and, recurrence of low back pain [3–7]. In general, patients with CLBP show reduced maximum angle (MA) as well as reduced proprioception compared to pain-free persons [8]. Although not finally delineated, these movement alterations may be associated with impaired movement control; consequently, patients with CLBP may often display impaired trunk movement control [9–11]. Although the interactions of symptoms and trunk kinematics is not yet fully delineated, improving movement control might be a promising treatment goal during chronic non-specific low back pain rehabilitation.

Processes of motor learning are involved during improvements of movement control and movement patterns. The need of external feedback is evident to activate motor learning processes and to enhance motor performance [12]. Thus, new (health) (bio)feedback technologies are a promising approach by encouraging an external focus during movement [10, 13–15]. Such health technologies, based on movement sensors and augmented performance feedback, are able to provide reliable real-time feedback in combination with game-based task and therefore are favorable for this therapy approach [10, 13, 16–18]. A positive effect of sensor-based feedback compared to mirror- or therapist-based feedback was shown in the past [10]. Matheve et al. [19] proofed a hyperalgesia effect even after a single bout of game-based exercise intervention. However, the effect on trunk movement and motion control in CLBP patients remains unclear.

Exercise is effective for CLBP therapy and rehabilitation [20–22]. Specifically, the effects of motor control exercise therapies are highlighted in various meta-analyses on CLBP: reduction of pain and disability, as well as improvement in trunk movement control are evident [20, 21, 23–25]. Key components of motor control exercises are musculoskeletal control through afferent sensory/proprioceptive input, central nervous integration of afferents, and optimal stabilization to ensure functional dynamic joint stability in perturbative situations [25, 26]. Already a small number of repetitions may be sufficient to improve motor learning processes, i.e., movement performance and control, in healthy individuals [27]. Both, the training principle itself and the small number of repetitions needed (or even single bout-effects), have not yet been sufficiently investigated in those with CLBP but may be promising in improving motor control.

One of the limitations of conventional therapy is the lack of objective and real-time feedback on movement quality and execution. In addition, traditional exercises are associated with a high intrinsic loss of motivation over time. This often leads to a low compliance to the prescribed and evident intervention as reported in numerous studies [13, 28]. To enhance compliance to the therapy, sensor-based exergames can be a promising training mean in CLBP therapy and rehabilitation to improve reliable real-time feedback and allow a time- and place independent training over a longer period of time [13, 14]. Zadro et al. [14] proofed an increased adherence to the prescribed exercise time (> 70%) in elderly people with CLBP by applying an exergame [14]. Nevertheless, it remains unclear if already a single bout of a game-based real-time feedback intervention can be effective in the treatment of chronic low back pain and trunk movement control.

Therefore, the purpose of the presented study was to evaluate the acute effects of a single bout of game-based real-time feedback intervention on three-dimensional trunk movement in patients with chronic non-specific low back pain. It is hypothesized that a single bout of a game-based biofeedback intervention enhances trunk movement control in patients with CLBP, represented as an increased total range of motion as well as an increased angle reproduction capacity during lateral flexion. This study may show that a single bout of game-based real-time feedback intervention may already have a positive effect on movement control in patients with chronic non-specific low back pain. Following this, this form of intervention could be a tool of choice for physicians and therapists to acutely help low back pain patients.

## Material and methods

### Design and ethics

**Fig. 1:**
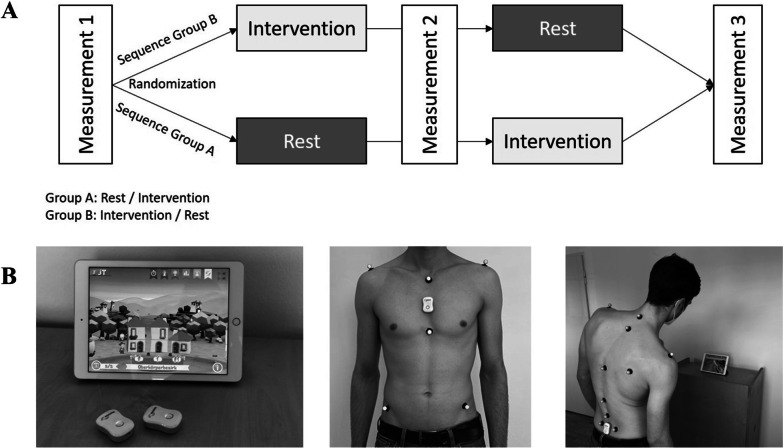
**A** Study protocol chart. **B** Medical device for digital back therapy (Valedo Home; Hocoma, Switzerland)

The presented study was conducted as a randomized cross-over trial with two arms (intervention/rest-period) in accordance with the recommendations of the “CONSORT 2010 statement: extension to randomised crossover trials” [29]. Each participant had one measurement session of about 2 h in a laboratory setting including three kinematic measurements (M1, M2, M3) and the two periods (rest/intervention) in between (Fig. 1A). The local Ethical Commission approved the study including all the described procedures (No. 10-2019). The trial was retrospectively registered in the German Clinical Trial Register (No. DRKS00029765; date of registration 27.07.2022).

### Participants

The sample size calculation was done using G*power (version 3.1.9.2, Düsseldorf, Germany) for a repeated measures univariate analysis of variance. Expecting a minimal effect size (f) of 0.3, assuming an alpha error of 5% and a beta-error of 80% n = 13 participants must be measured when a repeated measures default correlation of r = 0.7 is suggested [13].

Thus, n = 13 participants were recruited; in the professional environment (physiotherapy practice) of the principle investigators (JM, LO, AN). Inclusion criteria were an age from 18 to 70 years and existing chronic non-specific low back pain (≥ 3 month), diagnosed by a physician. Participants were excluded from study participation when representing high acute low back pain (≥ 5 at a visual analogue scale (VAS) of 0–10 cm in the last seven days), diagnosed with specific back pain (e.g. fracture; herniated disc), and/or pregnancy.

The principle investigator ascertained the eligibility of participants according to the inclusion and exclusion criteria. Before starting the measurements, all participants were informed about the procedure, aim and risks of the study as well as the possibility to terminate the participation in the study at any time, without giving reasons. Then, all voluntarily signed a written informed consent form.

### Randomization

Eligible participants were randomized (full randomization) in a 1:1 allocation to one of two treatment sequences-intervention-control or control-intervention. The randomisation list, generated by “randomization.com”, was kept in a locked cabinet. A research assistant not involved in the outcome assessment revealed the group allocation. All participants were blinded against the group sequence.

### Intervention

The game-based real-time biofeedback training was performed using a medical device for digital (home-based) back pain therapy (Valedo Home; Hocoma, Switzerland; Fig. 1B). This system consists of two inertial measurement sensors as well as the application-based software and a tablet/smartphone. The concept of the digital back therapy is that game-based exercises for movement control and stabilization of the trunk muscles are performed by the patient. With the help of the two sensors, the patient guides and controls an avatar via body movement through different virtual worlds.

To start the intervention, the two sensors were positioned over the lower lumbar spine as well as the sternum during upright standing (Fig. 1B). Afterwards the sensors were calibrated and the individual range of motion was measured. The intervention itself started with a one-minute trunk stabilisation task during two-legged standing. This was followed by a magic mirror task, were the patient needs to imitate five given movements of the trunk/pelvis. Afterwards the movement game for lateral flexion followed. The patient had to move an avatar with focus on right- and left-sided lateral flexion in three different levels. In total, the intervention last 12 min. The resting phase comprised the same period of time.

### Procedure

After receiving an anthropometric (body height (cm); body mass (kg)) assessment, all participants completed a paper–pencil-based version of the graded chronic pain questionnaire (von Korff), valid to measure the presence of chronic low back pain [30–32]. In addition, low back pain intensity was monitored during the entire protocol.

Participants were then prepared for kinematic analysis of trunk motion (see kinematic analysis). Afterwards, trunk motion during lateral flexion (while upright standing) was measured. Lateral flexion movement is a represent for one-sided trunk movement in daily living, e.g. one-handed lifting of a bag or box [33, 34]. In addition, it’s an isolated, controlled movement of the trunk in one plane avoiding high impact on the trunk of the patient’s analysed. Therefore, maximum angle (to the left and right side) and the individual angle reproduction were measured in a randomized order. For the assessment of maximum angle (MA), all participants performed one preparation and three test repetitions of maximum left- and right sided lateral flexion during upright standing. For angle reproduction, a target trunk angle of 20° in lateral flexion was set, as this is the reference value for lateral trunk flexion according to the neutral-zero-method. During the preparation trials, the target angle of 20° was positioned manually by a physiotherapist by use of a handheld goniometer placed on the back of each participant (baseline set at pelvis level). Each participant performed two preparation trials and ten test trials in each direction (right/left).

In between the three (kinematic) measurements, the 12-min intervention and the 12-min-rest-time was undertaken in random order; no additional wash out time was included.

### Kinematic analysis

**Fig. 2:**
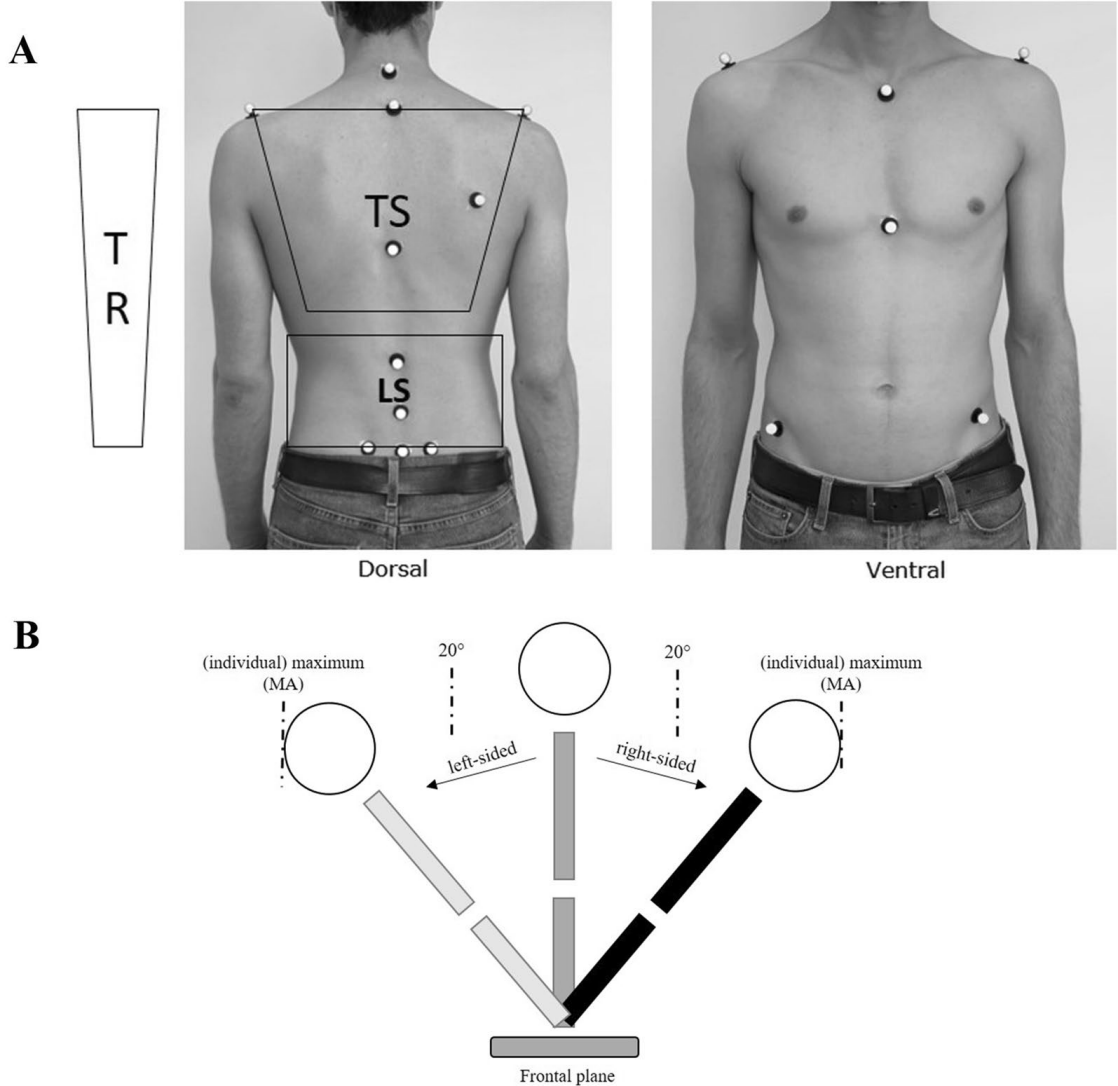
Visualization of kinematic trunk movement analysis. **A** Kinematic trunk model (Legend: *TS* thoracic segment, *LS* lumbar segment, *TR* trunk segment) here. **B** Kinematic trunk movement of lateral flexion (Legend: MA)

All kinematic trunk motion outcomes were measured using a 16-camera optoelectronic 3D-motion analysis system (Optitrack, Oregan, USA; 120 Hz). For tracking multi-segment trunk kinematics, 15 markers were positioned over bony landmarks to frame two segments (thoracic segment (TS), lumbar segment (LS)) (Fig. 2A) [35]. A total of ten markers were placed on the torso; four markers were placed around the pelvis, and an additional back marker was added on the right scapula especially for improved identification of left and right side of the tracked skeleton. In addition, the whole trunk segment was analysed as the sum of the two segments (trunk segment (TR)).

Marker data were analyzed to calculate the relative angles of each segment in relation to the pelvis. Therefore, the software-based filtered data (5 Hz cut off; Motive 2.0, Optitrack, Oregan, USA) were exported to a customized software (Python, Trier University of Applied Sciences) for calculation of the main measures (maximum angle [°], movement duration [s], movement velocity [°/s]).

### Primary outcomes

The primary outcome measurement was the maximum angle (MA; [°]) during left- and right sided maximum lateral flexion movement while upright standing. The secondary outcome was the individual normalized angle reproduction (AR; [°]) to a defined target movement angle in lateral flexion movement (Fig. 2B).

The maximum angle was assessed for lateral flexion as well as anterior flexion and axial rotation of each segment [36, 37]. The MA was measured as the maximum angle in all three planes while performing a maximum right-/left-sided lateral flexion described with good to excellent reliability [38]. Positive values represent trunk anterior flexion, right-sided lateral flexion and left-sided rotation. In contrast, negative values characterize trunk extension, left-sided lateral flexion and right-sided rotation.

In addition to the analysis of the MA, the angle reproduction was analysed for trunk lateral flexion described valid in low back pain patients [39]. The target angle for the reproduction task was set to 20° related to activities of daily life. To account for the manual positioning of the 20° target angle during the preparation trials by use of a handheld goniometer, the mean value out of the two preparation trials was calculated as a reference value for the test trials. The results of the ten (test) repetitions were then normalized to this mean value of the preparation trial as follows: the differences between the individual mean of the preparation trials and the ten repetition trials were calculated. In a final step, the individual mean values between preparation trial mean and the ten repetitions were calculated and are the basis for the pre/post comparison in the statistical analysis.

### Secondary outcomes

Further secondary (kinematic) outcomes were movement velocity [°/s] and duration [s]. Both were calculated for the whole movement cycle from upright standing to maximum (left- or right-sided) lateral flexion and back to upright standing.

#### Graded pain questionnaire

The graded pain questionnaire consists of seven items, including pain intensity and disability (recently and last 3 months) [6, 30–32]. Six items are conducted of a numeric rating scale ranging from 0 (no pain/disability) to 10 (highest pain/disability (incapable of doing anything)). Sub-scores of pain and disability are calculated. Furthermore, participants can get classified into one of the five hierarchical pain and disability grades ranging from low pain/disability (grade 0) to high pain/disability scores (grade IV) [6, 30–32].

#### Visual analogue scale

In addition to the graded pain questionnaire, low back pain was monitored by use of a VAS (0–10 cm) in regular intervals throughout the whole measurement day (begin/after each measurement/after intervention or rest/end). A score higher seven at the VAS (acute pain) was defined as termination criterion for the whole measurement.

### Data processing and statistical analysis

All non-digital data were documented in a paper and pencil-based case report form and transferred to the statistical database (JMP Statistical Software Package 14, SAS Institute®)[6]. After the plausibility check (range check and extreme/outlier value analysis for all outcomes), the data were presented descriptively (means, standard deviations, 95% confidence intervall) for all given outcomes [6].

All outcomes were checked for normal distribution with Shapiro–Wilk-Test [6]. Since the majority of the main outcomes were normally distributed, the following inferential statistical analysis was done two-stepped: Firstly, the measured data were analyzed for a potential carryover effect between the two periods (rest—intervention). Therefore, the sums of the measured outcome values after each period are calculated and an unpaired t-test for group comparison was applied [40].

Secondly, pre-to-post-intervention data were matched and analyzed by repeated measures analyses of variances to account for the potential acute interventional effects. The level of significance was set at α = 0.05 for all analyses.

## Results

### Flow of participants

**Fig. 3:**
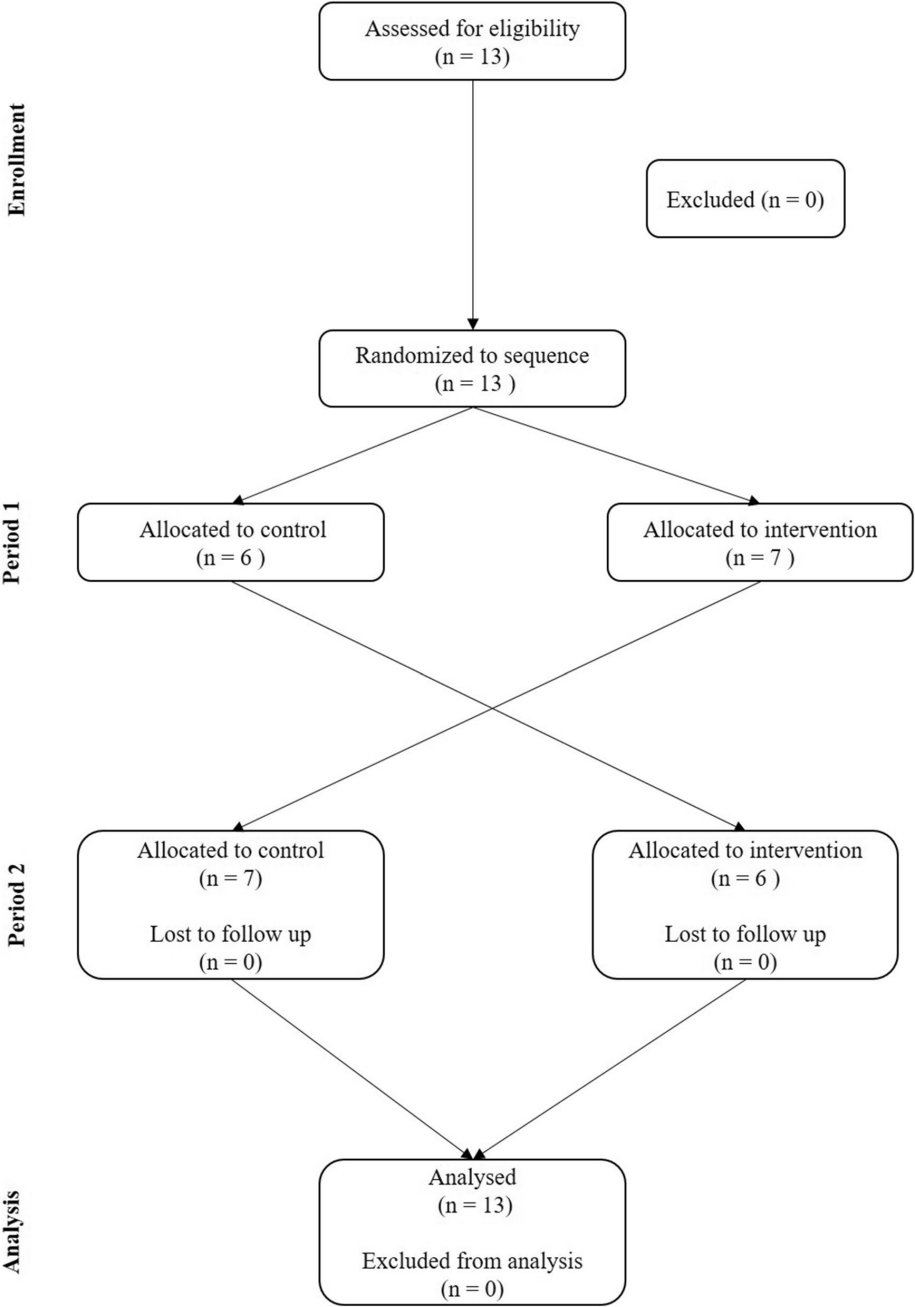
Flow chart of participants included into the study

From the included 13 participants with CLBP (eight female: 43 ± 8 years; 168 ± 8 cm height, 67 ± 7 kg body weight, LBP VAS 3.7 ± 2.7 cm; five male: 35 ± 20 years, 180 ± 7 cm height, 95 ± 28 kg body weight, LBP VAS 2.7 ± 2.4 cm), no one withdrew consent, no one was excluded (Fig. 3). No adverse effects, harms, or unintended effects occurred during the study conduction. Six participants (three male: 41 ± 25 years, 177 ± 3 cm height, 95 ± 21 kg body weight, LBP VAS 4.0 ± 2.2; three female: 34 ± 11 years, 168 ± 6 cm height, 71 ± 10 kg body weight, LBP VAS 2.6 ± 1.2) were allocated to the sequence control-intervention (group A), seven participants (two male: 26.5 ± 4 years, 184 ± 12 cm body height, 93 ± 47 kg body weight, LBP VAS 0.8 ± 1.1; five female: 50 ± 13 years, 168 ± 10 cm body height, 65 ± 10 kg body weight, LBP VAS 4.4 ± 3.2) vice versa (group B). There was no statistically significant difference in any of the baseline characteristics between the groups (p > 0.05).

### Pain

The results of the pain assessment (Graded Pain Questionnaire; VAS) are detailed in Table 1. Overall, no statistical significant reduction of CLBP (VAS) was measured comparing the pain assessment at the beginning and the end of the whole measurement session (p > 0.05). Moreover, the pain analysis directly pre and post intervention (exergame) revealed no significant differences (p > 0.05), too. Besides, one participant reported a pain score > 7 points after the first measurement (8.6 cm VAS). The participant was informed about the possibility of stopping the measurement. However, this participant especially wanted to continue the measurement voluntarily and all data were included into final data analysis.

**Table 1:** Low back pain intensity, disability, and classification represented as mean ± standard deviation

Group	Graded pain questionnaire outcomes			VAS (0–10 cm)	
	Pain score (0–100)	Disability score (0–100)	Classification grade (0–4)	Begin*	End*
All	46 ± 20	32 ± 16	2 ± 1	3.3 ± 2.5	2.6 ± 2.5
Group A (n = 6)	49 ± 19	38 ± 16	2 ± 1	3.3 ± 1.8	3.5 ± 2.4
Females (n = 3)	49 ± 8	34 ± 17	2 ± 1	2.6 ± 1.2	2.6 ± 2.3
Males (n = 3)	50 ± 30	42 ± 30	3 ± 2	4.0 ± 2.2	4.3 ± 2.5
Group B (n = 7)	43 ± 22	27 ± 16	2 ± 1	3.4 ± 3.2	1.9 ± 2.6
Females (n = 5)	52 ± 17	31 ± 16	2 ± 1	4.4 ± 3.2	2.3 ± 3.0
Males (n = 2)	20 ± 10	15 ± 3	1 ± 0	0.8 ± 1.1	0.65 ± 0.6

*Of the whole measurement day

### Carryover-effect

The results of the analysis to account for a possible carryover effect are detailed in Table 2 for the main (MA) and secondary (AR, movement velocity; movement duration) outcome measures. For all outcomes no significant carryover effects were present for the right-sided lateral flexion (p > 0.05). In contrast, there are significant carryover effects for the left-sided lateral flexion: the analysis of the MA showed significant effects in the trunk rotation for all segments as well as for the movement duration (Table 2A).

**Table 2:** (A) Results of the carryover effect analysis for maximum angle, angle reproduction, movement velocity and movement duration, (B) Effect size for the results of maximum angle (MA; (°)) during left- and right-sided lateral flexion for both segments as well as the whole trunk segment (Cohen’s d)

(A) Results of the carryover effect analysis for maximum angle, angle reproduction, movement velocity and movement duration			
Outcome Maximum angle			
Segment	Plane	p-values	
		Right side	Left side
Thoracic segment	Flexion/extension	0.74	0.32
	Rotation	0.86	0.01*
	Lateral flexion	0.25	0.83
Lumbar segment	Flexion/extension	0.63	0.41
	Rotation	0.85	0.01*
	Lateral flexion	0.27	0.06
Trunk segment	Flexion/extension	0.23	0.50
	Rotation	1.00	0.01*
	Lateral flexion	0.12	0.11

Outcome Angle reproduction			
Segment	Plane	p-values	
		Right side	Left side
Thoracic segment	Lateral flexion	0.78	0.87
Lumbar segment	Lateral flexion	0.22	0.07
Trunk segment	Lateral flexion	0.24	0.13

Secondary kinematic outcomes		p-values	
		Right side	Left side
Movement velocity		0.07	0.03*
Movement duration		0.39	0.52

(B) Effect size for the results of maximum angle (MA; (°)) during left- and right-sided lateral flexion for both segments as well as the whole trunk segment (Cohen’s d)			
Outcome Maximum angle			
Segment	Plane	Cohen’s d	
		Right side	Left side
Thoracic segment	Flexion/extension	0.20	− 0.15
	Rotation	− 0.12	0.22
	Lateral flexion	− 0.03	0.08
Lumbar segment	Flexion/extension	0.20	− 0.07
	Rotation	0.04	0.22
	Lateral flexion	0.18	0.30
Trunk segment	Flexion/extension	0.48	− 0.20
	Rotation	− 0.06	0.23
	Lateral flexion	0.13	0.23

### Intervention effect

#### Maximum angle

**Fig. 4:**
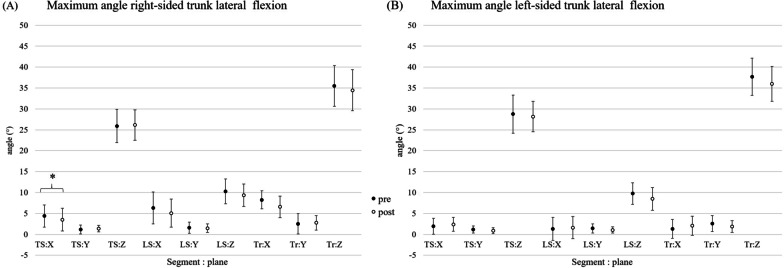
Pre and post intervention results (mean and 95% confidence interval) for MA (°) during right- (**A**) and left-sided (**B**) lateral flexion for both segments as well as the whole trunk segment. Legend: *TS* thoracic segment, *LS* lumbar segment, *Tr* whole trunk segment, *X* trunk extension/flexion, *Y* trunk rotation, *Z* trunk lateral flexion; *significant intervention effect

No significant change from pre to post intervention for MA (frontal-plane) for both segments as well as the whole trunk could be observed for right- and left-sided lateral flexion (p > 0.05) (Fig. 4). In contrast, the thoracic trunk segment showed a significant reduction of MA for trunk extension/flexion (sagittal plane; right-sided) from pre to post intervention (4.4° ± 4.4° (95% CI 7.06–1.75)/3.5° ± 1.29° (95% CI 6.22–0.80); p = 0.02) (Fig. 4; Table. 2B).

#### Angle reproduction

The analysis of the preparation trials showed a mean deviation of < 2° during the preparation trials to the target angle of 20° (see Table 3). This verifies the individual normalization of the angle reproduction task to the individual preparation trials. Results for the individual normalized angle reproduction are detailed in Table 3, likewise. No significant change from pre to post intervention in lateral flexion (z plane) for both segments as well as the whole trunk could be found (p > 0.05).

**Table 3:** Results of the angle reproduction (A) and movement velocity/duration (B) analysis represented by mean, standard deviations (SD), 95% confidence interval (CI), p-values and effect size (Cohen’s D)

(A) Angle reproduction											
Segment: Plane	Outcome	Pre (°)				Post (°)				p-value	Cohen’s d
		Mean	SD	Upper 95% CI	Lower 95% CI	Mean	SD	Upper 95% CI	Lower 95% CI		
Trunk segment: lateral flexion	P_mean_	18.66	5.85	22.20	15.12	19.81	5.60	23.19	16.43	0.62	− 0.20
Trunk segment: lateral flexion	AR	− 2.88	3.14	− 0.98	− 4.78	− 3.17	2.46	− 1.68	− 4.65	0.65	0.09

(B) Movement velocity and movement duration											
Outcome		Pre (°)				Post (°)				p-value	Cohen’s d
		Mean	SD	Upper 95% CI	Lower 95% CI	Mean	SD	Upper 95% CI	Lower 95% CI		
Movement velocity (°/s)		4.61	1.59	5.57	3.65	4.08	1.52	5.00	3.17	0.13	0.33
Movement duration (sec)		4.83	1.05	5.46	4.20	4.87	1.12	5.54	4.19	0.88	− 0.04

Legend: P_mean_: mean value of the maximum angle for the two preparation trials (right side); AR: individual normalized angle reproduction (mean difference between P_mean_ − maximum angle of repetition 1–10)

#### Movement velocity and movement duration

The analysis of the movement velocity as well as the movement duration revealed no significant pre-to-post-intervention effect for left- and right-sided lateral flexion movement cycle (p > 0.05) (Table 3).

## Discussion

The main goal of the study was to assess the acute effects of a single bout of game-based real-time feedback intervention on 3D trunk movement in patients with CLBP. It was hypothesized that a single bout of a game-based biofeedback intervention enhances trunk movement control in patients with CLBP, represented as an increased total range of motion as well as an increased angle reproduction capacity during lateral flexion. However, no significant change from pre-to-post intervention for maximum angle as well as the individual normalized angle reproduction for right- and left-sided lateral flexion movement occurred. The thoracic trunk segment showed a significant reduction of the maximum angle for maximum angle in the sagittal plane (trunk extension/flexion) from pre to post intervention during the right-sided lateral flexion movement. In addition, no significant effect for pain analysis could be proven.

Although no acute intervention effect could be found for trunk motion analysis in the main movement plane (lateral flexion), the alterations of the secondary movement planes might indicate reduced evasive motion (rotation; flexion/extension). This may represent improved trunk motion control in patients with CLBP even after a single bout of a game-based real-time biofeedback intervention. This is in line with results from Matheve et al. [10], who could also proof significant effects of a single bout of sensor-based biofeedback session on trunk movement in patients with CLBP. However, the analysis of Matheve et al. [10] focused on the movement control in the sagittal plane. In addition, Luomajoki et al. [18] reported in a systematic review that movement control exercise is effective to reduce disability in people with chronic non-specific low back pain.

Lateral flexion movement was used as a representative for one-handed lifting task of the trunk during daily live [41]. Trunk extension/flexion movement, as a representative for two-handed lifting tasks, is very well investigated in asymptomatic persons and patients with CLBP [41, 42], even in the clinical setting and medical examination of CLBP. Since lifting movements are omnipresent in everyday life, they can be described as automated movement patterns [43, 44]. In comparisons of one-handed to two-handed lifting tasks, it becomes obvious that two handed lifting with a focus on trunk flexion can be discussed as a more stable, automated pattern [41, 42]. In contrast, one-sided lifting with focus on trunk lateral flexion movement reflects a more variable movement scenario represented by reduced movement reliability and higher demands on trunk movement control for the participants [6]. Moreover, reliability of the trunk lateral flexion measurements (kinematic analysis) has been described as reduced in previous investigations [35, 45, 46] during trunk extension/flexion tasks. Based on the already well investigated knowledge on trunk movement in CLPB in the sagittal plane, lateral flexion movement was chosen as the primary motion task in our study due to the higher demands on trunk movement control for the patients. According to the presented results, it can be speculated that the secondary movement planes (here extension/flexion and rotation) guarantee functional variability for the control and regulation of a straightforward primary movement direction of the trunk (here lateral flexion movement), to ensure stability of the movement task. In contrast, the primary movement direction (lateral flexion) represents a stable pattern and therefore did not show any changes [43, 44, 47]. Todorov and Jordan [47] discuss this observation as an optimised movement strategy that uses increased motion variability in the less relevant secondary movement directions, aiming to optimize the motor control of a segment. Consequently, the absence of kinematic differences between pre and post intervention analysis during lateral flexion in the primary movement plane can be discussed as a result of an automated movement pattern, even in CLBP patients.

Consistent with recent research [13, 19], the applied game-based exercise intervention is feasible in the context of low back pain patients. Therefore, the use of this game-based exercise interventions with real-time feedback can be applied to patients with chronic non-specific low back pain to distract from pain while performing exercise [13, 19]. Besides, no significant intervention effect for pain analysis could be shown which is in contrast to a recent study reported by Matheve et al. [19]. However, a trend towards pain reduction could be observed. A larger sample size might provide significant results in future studies. The non-significant result can be attributed to the fact that not all individuals react the same to exercise (acutely). Whilst, usually, an exercise-induced hyperalgesia is given (also acutely) [19, 48], some participants also suffer from an acute symptom worsening, which usually releases shortly after/directly after exercising [49]. No subjects dropped out during the study, although one patient reported more severe back pain (VAS > 7) after the first trunk measurement. However, this participant wanted to continue the study participation voluntarily, the pain decreased again in the further course of the measurements. One reason for the increase of pain may have been the high level of pain already before the start of the study (VAS = 7 start of the measurement session). However, the feasibility of this intervention have been proven elsewhere [13]. Besides, the presented results need to be interpreted with care due to the small sample size analyzed. Our results must thus be interpreted as explorative pilot findings and should be proven or disproven by future studies. Further, the lack of considering a real control condition in the statistical analysis must be seen as a limitation. Results can further only be representative for single interventional bouts and effects. A carryover effect for the left-sided trunk lateral flexion movement in the rotational plane (y plane) was seen. One may conclude that the results for the left-sided trunk lateral flexion movement in the rotational plane may be treated with more caution than the main results. A longer washout phase might had reduced this effect. Moreover, the integration of game-based biofeedback in therapy routines are challenges that need to be addressed in the future. Some participants had coordination problems when controlling the avatar by their own trunk movement, which were not completely eliminated even by a short test phase before the start of the intervention. Therefore, a longer phase to familiarize with the movement and the implementation in the digital game is recommended. Hence, the duration of the intervention session (12 min) might therefore be chosen too short overall to make already short-term effects fully visible.

## Conclusions

A single bout of game-based real-time feedback intervention lead to changes in the secondary movement planes indicating reduced evasive motion during trunk movement in chronic low back pain patients. Improved trunk motion control in patients with CLBP could be seen even after a single bout of a game-based real-time biofeedback training. Future studies should investigate (controlled setting) whether single sessions of a game-based real-time feedback intervention have a significant effect on trunk movement and pain reduction in patients with CLBP who report a high pain level or are in a current phase of severe pain. The focus could be to investigate whether these patients can be helped with already one session, which could be a useful addition to the daily therapeutic routine.

## Data Availability

Main data generated or analysed during this study are included in this published article. Furthermore, the datasets used and/or analysed during the current study are available from the corresponding author on reasonable request.

## Abbreviations

AR: Angle reproduction
CLBP: Chronic non-specific low back pain
LBP: Low back pain
LS: Lumbar segment
MA: Maximum angle
TR: Trunk segment
TS: Thoracic segment
VAS: Visual analogue scale
